# The Emerging Role of Neuropeptides in Parkinson’s Disease

**DOI:** 10.3389/fnagi.2021.646726

**Published:** 2021-03-08

**Authors:** Yanan Zheng, Linlin Zhang, Junxia Xie, Limin Shi

**Affiliations:** ^1^Department of Physiology, Shandong Provincial Key Laboratory of Pathogenesis and Prevention of Neurological Disorders, School of Basic Medicine, Qingdao University, Qingdao, China; ^2^Institute of Brain Science and Disease, Qingdao University, Qingdao, China

**Keywords:** Parkinson’s disease, neuropeptides, ghrelin, neuropeptide Y, pituitary adenylate cyclase-activating polypeptide, substance P, neurotensin

## Abstract

Parkinson’s disease (PD), the second most common age-related neurodegenerative disease, results from the loss of dopamine neurons in the substantia nigra. This disease is characterized by cardinal non-motor and motor symptoms. Several studies have demonstrated that neuropeptides, such as ghrelin, neuropeptide Y, pituitary adenylate cyclase-activating polypeptide, substance P, and neurotensin, are related to the onset of PD. This review mainly describes the changes in these neuropeptides and their receptors in the substantia nigra-striatum system as well as the other PD-related brain regions. Based on several *in vitro* and *in vivo* studies, most neuropeptides play a significant neuroprotective role in PD by preventing caspase-3 activation, decreasing mitochondrial-related oxidative stress, increasing mitochondrial biogenesis, inhibiting microglial activation, and anti-autophagic activity. Thus, neuropeptides may provide a new strategy for PD therapy.

## Introduction

Parkinson’s disease (PD) is a progressive age-related neurodegenerative disease that results in cardinal motor symptoms, such as bradykinesia, muscle rigidity, and static tremor. The neuropathogenesis of PD is characterized by selective lesions of dopamine (DA) neurons in the mesencephalic substantia nigra (SN)–striatum pathway and the formation of Lewy bodies in the remaining neurons (Obeso et al., 2000). DA replacement therapies (L-DOPA) can relieve these symptoms; however, their prolonged use often causes severe side effects, marked by involuntary muscle movements.

Neuropeptides are small protein-like molecules that are generated and secreted, though not solely, by neurons in the central and peripheral nervous systems (Carniglia et al., 2017). Most neuropeptides act on G-protein coupled receptors (GPCRs). Peptide-GPCRs signals are known to be involved in various brain functions, such as glucose metabolism, learning and memory, stress and anxiety, food intake, reward, sleep and wakefulness, and neuroprotection (Jiao et al., 2017; Chen et al., 2018). Recently, several neuropeptides such as ghrelin, neuropeptide Y (NPY), pituitary adenylate cyclase-activating polypeptide (PACAP), substance P (SP), and neurotensin have been shown to play neuroprotective roles in PD both *in vivo* and *in vitro* (Wang et al., 2015b; Bayliss et al., 2016a; Maasz et al., 2017; Shi et al., 2017; Lazarova et al., 2018; Li et al., 2019). In this review, we discuss the relationship between these neuropeptides and PD and their underlying mechanisms. Additionally, patients with PD exhibit aberrant expression of central and peripheral neuropeptides. The changes in PD-related neuropeptides levels are also discussed, which might provide a possible basis for the development of a putative biomarker for the diagnosis and management of PD.

## Ghrelin

Ghrelin, a 28-amino-acid protein, acts as an exclusive endogenous ligand for the growth hormone secretagogue receptor 1a (GHS-R1a). It is mainly secreted in the stomach and is expressed in the pituitary, the internuclear space between the arcuate nucleus, the lateral hypothalamus, the dorsomedial nucleus, the ventromedial nucleus, the ependymal layer of the third ventricle, and the paraventricular nucleus (Kojima et al., 1999; Cowley et al., 2003; Hou et al., 2006). Two circulating forms of ghrelin exist in the plasma: acylated and deacylated ghrelin (Bayliss et al., 2016a). Acyl-ghrelin is believed to activate the GHS-R1a to exert biological effects (Delhanty et al., 2012). Previous studies have determined that the acylated form is responsible for neuroprotection in PD (Bayliss et al., 2016a; Wagner et al., 2017).

Downregulated levels of ghrelin and GHS-R1a are known to be closely related to the pathogenesis of PD. In patients with PD, the fasting levels of both active (acylated form) and total ghrelin were downregulated, with a relatively substantial decrease in the active form, compared with healthy controls (Table 1; Song et al., 2017). The genetic ablation of GHSR was found to aggravate the decrease in nigral DA neurons and lower striatal DA levels in PD animal models, which could be reversed by selectively reactivating GHSR in catecholaminergic neurons (Andrews et al., 2009). Additionally, other studies revealed that either an intracerebroventricular injection or microinjection of the selective GHS-R1a antagonist [D-Lys3]-GHRP6 into the SN of normal mice could produce PD-like dysfunction in motor coordination (Suda et al., 2018).

**TABLE 1:** Changes of neuropeptides and receptors in PD.

Neuropeptides/Receptors	Changes	Biological sample	State of disease		References
			
			Human	Animal models	
Ghrelin	↓	Plasma	PD patients		Song et al., 2017
Neuropeptide Y	↓	Adrenal medullary tissues	PD patients		Stoddard et al., 1991
Neuropeptide Y	↓	Cerebrospinal fluid	PD patients		Martignoni et al., 1992
Neuropeptide Y mRNA	↑	Caudate nucleus, putamen and nucleus accumbens	PD patients		Cannizzaro et al., 2003
Neuropeptide Y positive cells	↓	Caudate nucleus and putamen	X-linked dystonia-parkinsonism patients		Goto et al., 2013
Neuropeptide Y	↑	Striatum		MPTP-induced mouse model	Obuchowicz et al., 2003
PAC1 receptor	↓	Caudate nucleus, putamen, and globus pallidus		MPTP-induced macaque models	Feher et al., 2018
Substance P	↓	SN and the external segment of the globus pallidus	PD patients		Mauborgne et al., 1983
Substance P	↓	Saliva	PD patients		Schroder et al., 2019
Substance P	↓	SN and striatum		6-OHDA-induced PD rat model (3–4 weeks after lesion)	Lindefors et al., 1989
Substance P	↑	SN		6-OHDA-induced PD rat models (3–21 days after lesion)	Thornton and Vink, 2012
Neurotensin	↑	SN	PD patients		Fernandez et al., 1995, 1996
Neurotensin	↑	Plasma	PD patients		Schimpff et al., 2001
NT receptors mRNA	↓	the ventral tier of the substantia nigra	PD patients		Yamada et al., 1995
NT receptors	↓	Putamen and globus pallidus	PD patients		Uhl et al., 1984; Chinaglia et al., 1990; Fernandez et al., 1994

Our group was the first to report the neuroprotective effects of ghrelin in an 1-methyl-4-phenyl-1,2,3,6-tetrahydropyridine (MPTP)-induced PD model (Jiang et al., 2008), which were later confirmed by other studies (Andrews et al., 2009; Moon et al., 2009; Yu et al., 2016). Ghrelin counteracted rotenone-induced cell loss (Yu et al., 2016; Liu et al., 2018), improved the impaired performance of rota-rod in the mouse MPTP-induced model of PD (Moon et al., 2009), and mediated the neuroprotective effects of caloric restriction (Bayliss et al., 2016b). Additionally, ghrelin has been shown to electrically activate DA neurons via the inhibition of KCNQ and A-type potassium channels and upregulate HCN channels to improve the inhibition of MPP^+^ on the excitability of DA neurons (Shi et al., 2013; Chang et al., 2020; Xue et al., 2020). The most recent research showed that ghrelin enhanced the proliferation, migration, and differentiation of midbrain neural stem cells via the Wnt/β-catenin pathway, which proposed a novel possibility that ghrelin might be clinically valuable for the treatment of PD (Gong et al., 2020). Additionally, chronic treatment with ghrelin agonist HM01 was found to improve 6-OHDA lesion-induced non-motor symptoms in a rat model of PD, including alterations in body weight, fecal weight, food intake, and water consumption (Minalyan et al., 2019). Ghrelin analog, Dpr3ghr, also protected SH-SY5Y cells from methylglyoxal-induced neurotoxicity and apoptosis (Popelova et al., 2018).

**FIGURE 1:**
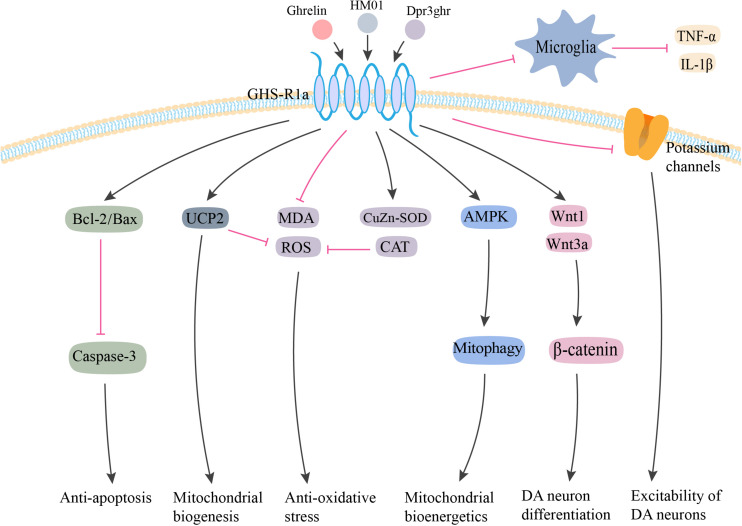
Potential neuroprotective effects of ghrelin in PD. Ghrelin and its analog produce neuroprotective effects against MPTP/MPP^+^ and rotenone-induced toxicity via anti-apoptosis, anti-oxidative stress, and anti-inflammation. By interacting with GHS-R1a, ghrelin inhibits neuronal apoptosis via increasing Bcl-2/Bax ratio and reducing Caspase-3 activation. Ghrelin enhances the antioxidant enzyme CAT and CuZn-SOD expression, and inhibits the lipid peroxidation and ROS production. The upregulation of UCP-2 caused by ghrelin could also buffer ROS production, as well as promote mitochondrial biogenesis. Additionally, ghrelin prevents inflammatory microglial activation and enhances neuronal excitability by inhibiting voltage-gated potassium channels. Ghrelin also has the capability to enhance the proliferation, migration, and differentiation of midbrain neural stem cells via the Wnt/β-catenin pathway. Furthermore, ghrelin is proposed to provide neuroprotective effects via AMPK activation and enhanced mitophagy to ultimately enhance mitochondrial bioenergetics. GHS-R1a, growth hormone secretagogue receptor-1a; UCP2, uncoupling protein 2; MDA, malonaldehyde; ROS, reactive oxygen species; CuZn-SOD, CuZn superoxide dismutase; CAT, catalase; AMPK, 5′ adenosine monophosphate-activated protein kinase; IL-1β, interleukin-1β; TNF-α, tumor necrosis factor-α; DA, dopamine. The red line is inhibition and the black line is activation.

The mechanisms underlying the neuroprotective effects of ghrelin are complex (Figure 1; Morgan et al., 2018). The first study based on a sub-acute MPTP-induced mouse model of PD demonstrated that ghrelin-mediated neuroprotection might be related to a reduction in caspase-3-mediated apoptosis via the regulation of gene expression of Bcl-2 and Bax in the DA neurons in SN (Jiang et al., 2008). Another study showed that ghrelin antagonized rotenone/MPP^+^-induced neurotoxicity in MES23.5 cells and retinal ganglion cells by inhibiting the activity of mitochondrial respiratory chain complex I, eliminating reactive oxygen species (ROS) synthesis, stabilizing mitochondrial transmembrane potential (Δψm), and inhibiting caspase-3 activation (Dong et al., 2009; Yu et al., 2016; Liu et al., 2018).

Ghrelin-induced neuroprotection is dependent on mitochondria-related oxidative stress and mitochondrial biogenesis. Andrews et al. (2009) proposed that ghrelin-induced uncoupling protein 2 (UCP2)-dependent alterations in mitochondrial respiration and a bioenergetics status provision, which might make the DA neurons more resistant toward cellular stress. Liu et al. (2010) demonstrated that ghrelin exerted its antioxidant effects by increasing the activity of Cu-Zn superoxide dismutase (CuZn-SOD) and catalase (CAT), decreasing the concentration of malonaldehyde (MDA), and inhibiting NF-κB translocation. Another study indicated that ghrelin-induced neuroprotection was dependent on the activation of AMPK (5′ adenosine monophosphate-activated protein kinase) and enhanced mitophagy in DA neurons (Bayliss and Andrews, 2013). Ghrelin could exert its neuroprotective effects by inhibiting the activation of microglia and the subsequent release of IL-1β and TNF-α in an MPTP-induced mouse model of PD (Moon et al., 2009).

## Neuropeptide Y

Neuropeptide Y (NPY), a 36-amino-acid peptide, was first isolated in 1982 from the porcine brain (Tatemoto, 1982). NPY is unequally distributed across the brain, with higher levels in the hypothalamus, amygdala, hippocampus, and striatum (de Quidt and Emson, 1986). NPY receptors are classified as GPCRs, five of which have already been cloned from mammals: Y1, Y2, Y4, Y5, and Y6 receptors (Li et al., 2019). The Y1 receptor mRNA is distributed in all layers of most limbic and neocortical regions, striatum, caudate, putamen, and nucleus accumbens (Caberlotto et al., 1997, 2000). The Y2 receptor mRNA is found in different areas, including the cerebral cortex, striatum, the hippocampal formation, and the nucleus accumbens (Caberlotto et al., 1998, 2000). The Y4 receptor mRNA is primarily found in the thalamus, subthalamic nucleus, hypothalamus, amygdala, SN, and lesser expression in the corpus callosum, caudate nucleus, and hippocampus (Yan et al., 1996). High levels of Y5 receptor mRNA are found in the SN, hypothalamus, and amygdala (Nichol et al., 1999). The Y6 receptor mRNA is only functionally expressed in the rabbit and mouse and is not expressed in the rat (Starback et al., 2000).

Several animal and human studies have detected changes in NPY levels. Stoddard et al. (1991) reported lower NPY levels in the adrenal medullary tissues in PD patients. Later, Martignoni et al. (1992) measured NPY-immunoreactivity (NPY-ir) levels in the cerebrospinal fluid (CSF) of 10 patients with PD and found them lower than that in healthy individuals, which indicated a reduction in NPY release or increase in NPY turnover. Moreover, the number of NPY mRNA positive cells was markedly increased in the caudate nucleus, putamen, and nucleus accumbens in the post-mortem brain specimens of patients with PD (Cannizzaro et al., 2003). Additionally, studies have found a decrease in NPY-positive cells and a significant loss of nerve fibers in the putamen and caudate nucleus in X-linked dystonia-parkinsonism patients (Goto et al., 2013). In the subventricular zone, these patients also lacked NPY labeling, along with a significant loss of progenitor cells that expressed proliferating cell nuclear antigen (Goto et al., 2013). Svenningsson et al. (2017) recently reported higher levels of NPY in CSF of PD patients with comorbid depression compared with those with major depressive disorder. In animal models of PD, the degeneration of the nigrostriatal DA pathway caused a remarkable increase in NPY-expressing cells in the striatum (Kerkerian et al., 1986, 1988; Obuchowicz et al., 2003). All these results supported an association between NPY and PD.

**FIGURE 2:**
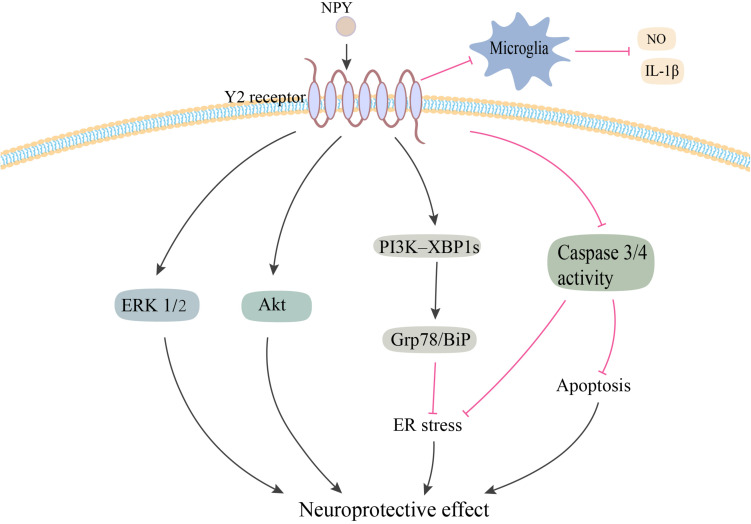
Potential neuroprotective effects of NPY in PD. NPY may be exerting its neuroprotective effect by directly acting on DA cells via Y2 receptor. NPY activates the ERK and Akt pathways, and inhibits ER stress–induced neuronal cell death by activating the PI3K-XBP1s-Gip78/BiP pathway. NPY treatment also inhibits apoptosis by suppressing the activation of caspase-3 and -4. Additionally, NPY prevents the activation of microglia and the production of NO and IL-1β. NPY, neuropeptide Y; ERK1/2, extracellular signal-regulated kinase 1/2; ER, endoplasmic reticulum; NO, nitric oxide; IL-1β, interleukin-1β. The red line is inhibition and the black line is activation.

Neuropeptide Y has been demonstrated to exert its potent neuroprotective effects via a variety of pathways related to PD (Figure 2). Decressac et al. (2012) first reported that NPY exerted its neuroprotective effects in both *in vitro* and *in vivo* 6-OHDA-induced models of PD. Using pharmacological antagonist and mice knockout for Y2 receptors, it was shown that NPY exerted its neuroprotective effects by acting on DA cells and terminals via the Y2 receptor, which induced the activation of the ERK and Akt pathways (Decressac et al., 2012). NPY also inhibited the activation of microglia in SN and striatum of 6-OHDA rats, which mediated the anti-inflammatory effect of NPY in PD (Pain et al., 2019). Another study confirmed the inhibitory effect of NPY on LPS-stimulated NO synthesis and IL-1β secretion in the microglia (Ferreira et al., 2010). Additionally, several studies have demonstrated higher levels of endoplasmic reticulum (ER) stress in common neurodegenerative diseases, such as Parkinson’s, Huntington’s, and Alzheimer’s diseases (Matus et al., 2011). A recent study found that NPY exerted a protective effect against cell death in ER stress–induced neurons by activating the PI3K-XBP1s-induced Gip78/BiP pathway (Lee et al., 2018). NPY treatment also suppressed the activation of caspase-3 and caspase-4 in the ER stress response pathway (Lee et al., 2018).

It was hypothesized that the neuroprotective mechanism of NPY was probably related to the brain-derived neurotrophic factor (BDNF), which is a neurotrophin that promotes neuronal survival and differentiation (Sendtner et al., 1992). Reduced expression of BDNF has been postulated to cause the loss of nigral DA neurons in PD (Sendtner et al., 1992; Fumagalli et al., 2006). Until now, there are no published reports on the possible relationship between NPY and PD-related BDNF expression. However, in mouse models of Machado–Joseph disease, an inherited neurodegenerative disorder, NPY overexpression was found to alleviate motor deficits and neuropathology by increasing BDNF expression and reducing neuroinflammation (Duarte-Neves et al., 2015). Future studies need to explore the possible effects of NPY on BDNF expression in PD.

## PACAP

Pituitary adenylate cyclase-activating polypeptide belongs to the superfamily of glucagon/secretin/vasoactive intestinal polypeptides (VIP); it was first isolated from an ovine hypothalamus (Miyata et al., 1989). PACAP is widely expressed, particularly at high concentrations, in the hypothalamus, nucleus accumbens, bed nucleus of the stria terminalis, and SN in the mammalian brain (Chung et al., 2005; Vaudry et al., 2009). Additionally, the PACAP genes had >2.0-fold elevation of mRNA levels in ventral tegmental area (VTA) compared with SN DA neurons (Chung et al., 2005). Exposing α-synuclein overexpressing PC12 cells and rat primary ventral mesencephalic cultures to PACAP decreased vulnerability of both cell types to MPP^+^, suggesting that the upregulation of PACAP gene in VTA could be one of the factors responsible for the altered vulnerability (Chung et al., 2005).

Pituitary adenylate cyclase-activating polypeptide receptors, which belong to the family of GPCRs, include PAC1, VPAC1, and VPAC2 receptors. The mRNA encoding the PACAP receptors has been identified in the SN (Hashimoto et al., 1996). Additionally, a significantly reduced PAC1 receptor immunosignal was detected in the putamen, caudate nucleus, and internal and external parts of the globus pallidus in MPTP-induced macaque models of PD (Wang et al., 2005; Feher et al., 2018).

Several studies have shown the neuroprotective impact of PACAP in different PD models (Reglodi et al., 2011, 2017). PACAP was found to attenuate 6-OHDA-induced loss of DA neurons, improve behavioral deficits (Reglodi et al., 2004a), reduce severe acute hypokinesia (Reglodi et al., 2004b), and attenuate the decrease in DA levels (Maasz et al., 2017). PACAP could prevent the MPTP-induced dysregulation of protein synthesis and attenuate cognitive decline (Deguil et al., 2010). In rotenone-induced PD cell models, PACAP was found to decrease cellular apoptosis and facilitate the transformation of cell apoptosis from late stage to early stage (Wang et al., 2005). In SH-SY5Y cells, PACAP protected against salsolinol-induced toxicity by attenuating apoptosis and the associated chemical changes (Brown et al., 2013). In a prostaglandin J2 (PGJ2)-induced mouse model of PD, PACAP27 reduced the loss of DA neurons and motor deficits (Shivers et al., 2014).

**FIGURE 3:**
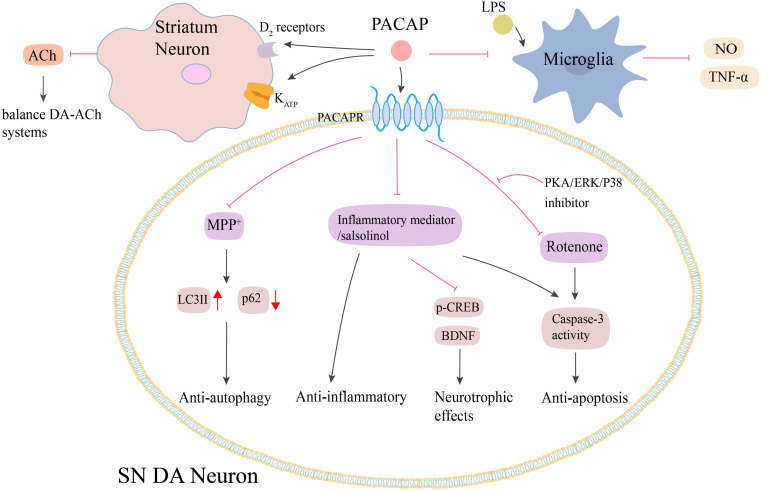
Potential neuroprotective effects of PACAP in PD. Both animal and cell models of PD show that PACAP produces neuroprotective effects against MPTP/MPP^+^, rotenone and salsolinol-induced toxicity via anti-autophagy, anti-inflammatory, and anti-apoptosis. PACAP diminishes MPP^+^-induced accumulation of LC3II and reduction of p62 protein. PACAP antagonizes salsolinol and rotenone-induced cell apoptosis by inhibiting the caspase-3 activity, which could be blocked by the administration of PKA, ERK, and p38 inhibitors. PACAP protects against inflammatory-mediated toxicity by enhancement of BDNF and its signal transduction protein p-CREB. Additionally, PACAP attenuates LPS-induced activation of microglia and the consequent NO synthesis and TNF-α secretion. PACAP also leads to the increase of D2 receptors activity and KATP subunits expression in the striatum, which results in the decrease of ACh secreting and finally regulates the balance between DA-ACh systems. PACAP, pituitary adenylate cyclase-activating polypeptide; PACAPR, pituitary adenylate cyclase-activating polypeptide receptors; LPS, lipopolysaccharide; NO, nitric oxide; TNF-α, tumor necrosis factor-α; MPP^+^, methyl-phenylpyridinium; p-CREB, phosphorylated cyclic adenosine monophosphate response element binding protein; BDNF, brain-derived neurotrophic factor; PKA, protein kinase A; ERK, extracellular signal-regulated kinase; SN, substantia nigra. The red line is inhibition and the black line is activation.

It has been shown that PACAP exerts its neuroprotective effects on PD via multiple mechanisms (Figure 3). First, PACAP-induced neuroprotection was associated with its anti-inflammatory effects. The pre-treatment of SH-SY5Y cells with PACAP (1–38) resulted in a dose-dependent attenuation of toxicity caused by the inflammatory mediators (Brown et al., 2014). Second, PACAP exhibited anti-autophagic properties. In the MPTP-induced models, PACAP reduced the autophagic activity by producing LC3II and modulating p62 protein levels (Lamine-Ajili et al., 2016). Third, Wang et al., 2005 found that the protective role of PACAP against rotenone-induced cell death was inhibited by the administration of PKA, ERK, and p38 inhibitors. Thus, PACAP exerted its neuroprotective effects by activating PKA signaling pathway as well as the downstream ERK and p38 MAPK signals (Wang et al., 2005). Fourth, this neuroprotective effect was found to be correlated to a balance between DA-ACh systems in the basal ganglia neuronal pathway. In the MPTP-induced PD mouse model, intravenous injection of PACAP27 offered neuroprotective effects by changing the cholinergic and dopaminergic neurotransmission, which were associated with the increase of the D2 receptors activity and KATP subunits expression in the striatum (Wang et al., 2008). Fifth, PACAP or its receptor agonists attenuated the salsolinol-induced toxicity of SH-SY5Y cells by enhancing the expression of BDNF and its signal transduction protein, p-CREB, and inhibiting the expression of caspase-3 (Brown et al., 2013). Apart from these mechanisms, PACAP-induced neuroprotection was probably associated with microglia. PACAP could attenuate LPS-induced activation of microglia and the consequent NO synthesis and TNF-α secretion (Yang et al., 2006). However, Shivers et al., 2014 reported that PACAP27 was unable to prevent microglial activation in the PGJ2-induced mouse model of PD (Shivers et al., 2014). Future studies need to explore the precise role of PACAP in microglial activation in PD.

Vasoactive intestinal polypeptides is a related peptide of PACAP which was first isolated from pig small intestine (Said and Mutt, 1970). Recent studies have found that it also has the similar neuroprotective effects as PACAP (Korkmaz and Tunçel, 2018). VIP prevented the MPTP-induced loss of DA neurons and nerve fibers in the nigrostriatal pathway by inhibiting of proinflammatory toxic molecules (i.e., TNF-α, ROS, NO, and IL-1β) (Delgado and Ganea, 2003). In 6-OHDA-induced PD rats models, VIP was found to reverse the rotational deficits, renovate myelin sheet (Tunçel et al., 2005), preserve corpus striatum neurons via producing nerve growth factor by brain mast cells (Korkmaz et al., 2010), exert the potential of anti-apoptosis and anti-oxidation to protect corpus striatum neurons by reducing DNA fragmentation and lipid peroxidation (Tunçel et al., 2012). Interestingly, Yu et al. (2020) found that VIP-TAT, which was similar to PACAP with two-dimensional structure, could increase more traversing potency than VIP to exert effective neuroprotective effect in the MPTP-induced PD mouse models.

## Substance P

Substance P (SP), an undecapeptide encoding the tachykinin 1 (TAC1) gene, belongs to the tachykinin family. It is known to exert multiple biological roles by acting on three different types of GPCRs called NK1, NK2, and NK3 receptors (Pennefather et al., 2004). SP is widely distributed throughout the central and peripheral nervous systems. High levels of SP are found in the basal ganglia, where the SN showed the highest levels (Kanazawa and Jessell, 1976).

In the 1980s, Mauborgne et al. (1983) found a significant decrease in SP-like immunoreactivity within the SN and the external segment of the globus pallidus in parkinsonian brains, which was later confirmed by other studies (Tenovuo et al., 1984; Sivam, 1991; De Ceballos and Lopez-Lozano, 1999). Compared with PD patients with normal pharyngeal swallowing function, significantly lower levels of SP in the saliva were found in those with pharyngeal dysphagia (Schroder et al., 2019). Other studies observed SP variations in 6-OHDA-induced PD rat models. An early study showed that the DA denervation decreased the concentration of SP in both SN and striatum (3–4 weeks after 6-OHDA lesion) (Lindefors et al., 1989). However, Thornton and Vink (2012) observed elevated nigral SP levels from days 3 to 21 after 6-OHDA treatment. It appeared that the 6-OHDA lesion resulted in an increase in SP levels initially followed by a decrease.

Until now, the effect of SP in the treatment of PD remains controversial. Wang et al. (2015b) found that SP protected MES23.5 cells from MPP^+^-induced cytotoxicity by decreasing calcium influx, regulating Δψm, and modulating ROS synthesis and caspase-3 activation. However, other studies have shown that SP exacerbates dopaminergic cell death. For example, in the 6-OHDA-induced model of PD, the administration of additional SP accelerated disease progression and exacerbated dopaminergic cell death, with animals displaying extensive motor deficits (Thornton and Vink, 2012). Exogenous SP activated microglia, followed by potentiating LPS- or MPP^+^-induced toxicity in primary dopaminergic cell cultures; the absence of endogenous SP in TAC1^–/–^ mice made them more resistant to neurotoxicity-induced dopaminergic neurodegeneration (Wang et al., 2014).

Similarly, both SP receptor agonists and antagonists demonstrated neuroprotective effects in PD. Septide [(Pyr6, Pro9)-SP (6–11)], the NK1 receptor agonist, ameliorated dopaminergic neurodegeneration and motor deficits via Akt/PKB signaling pathway in 6-OHDA-lesioned rats (Chu et al., 2011). Additionally, senktide, the NK3 receptor agonist, restored the temporal order memory in the animals (Chao et al., 2015). However, intracerebroventricular administration of the NK1 receptor antagonist L-733,060 or N-acetyl-L-tryptophan (NAT) also attenuated 6-OHDA-mediated cell death and resulted in a significant improvement in motor function (Thornton and Vink, 2012). NAT and another NK1 receptor antagonist, LY303870, reduced L-DOPA-induced dyskinesia without affecting the therapeutic role of L-DOPA in PD rats (Thornton et al., 2014; Yang et al., 2015).

Microglia mimics the role of macrophages in the brain; they are the primary form of active immune defense in the CNS. The substantia nigra pars compacta (SNc) contains a higher density of microglia than the surrounding brain regions (Kim et al., 2000). Recent findings have shown that SP might be partly responsible for the high density of microglia. Mice deficient in endogenous SP (TAC1^–/–^) or NK1R (NK1R^–/–^) showed a significant reduction in nigral microglial density (Wang et al., 2015a). Additionally, Wang et al. (2015a) illustrated that SP attracted microglia through a NK1R/PKCδ/NADPH oxidase pathway-dependent manner.

## Neurotensin

Neurotensin (NT), a 13-amino-acid peptide, was originally isolated from the bovine hypothalamus (Carraway and Leeman, 1973). It is closely involved in the dopaminergic system. Histological studies from rat brain have shown that abundant NT-containing fibers are found in DA-rich areas such as the ventral tegmental area and SN (Jomphe et al., 2006). Clinical research has shown a two-fold increase in NT concentration in the SN in the brain tissue specimens from patients with PD (Fernandez et al., 1995, 1996). The plasma NT concentration was also higher in drug-free PD patients compared with healthy controls and L-DOPA-treated subjects (Schimpff et al., 2001).

There are three subtypes of NT receptors, including NTS1, NTS2, and NTS3/sortilin-receptor (Vincent et al., 1999). High levels of NT receptor mRNA were found in rat brain DA neurons of the ventral tegmental area and SN (Yamada et al., 1995). However, in PD brain tissues, the ventral tier of the substantia nigra, very low levels or no expression of mRNA for NT receptors were found (Yamada et al., 1995). NT receptors levels were also markedly reduced in the putamen and globus pallidus of patients with PD (Uhl et al., 1984; Chinaglia et al., 1990; Fernandez et al., 1994).

Previous studies have shown that both NT and NT analogs exert neuroprotective effects in animal models of PD. Intracerebroventricular administration of NT8-13 and [D-Tyr11]-NT attenuated 6-OHDA-induced muscle rigidity and tremors (Rivest et al., 1991). Recent findings also demonstrated that two new NT analogs, NT2 and NT4, could significantly decrease the number of apomorphine-induced rotations, enhance DA release in the striatum, and improve learning and memory (Lazarova et al., 2018). However, according to Antonelli et al. (2007) NT increased the degeneration of cortical neurons and dopaminergic mesencephalic neurons by enhancing glutamate-induced neurotoxicity by increasing intracellular calcium and/or the amplification of the NMDA-mediated glutamate signaling. Further studies are required to clarify the possible role of NT in PD and dopamine systems.

## Conclusion

Here, we provided an overview of the recent studies on the role of neuropeptides, including ghrelin, NPY, PACAP, SP, and NT, in PD. Altered expressions of these neuropeptides and their receptors were found in PD-related regions, especially the SN-striatum pathway. Most neuropeptides exhibited neuroprotective effects against the selective lesion of DA neurons by inhibiting caspase-3 activation, attenuating mitochondria-related oxidative stress, inhibiting microglial activation, anti-inflammation, and anti-autophagic activity. Moreover, peptide analogs and receptor agonists or antagonists were also used to protect against neurotoxin-elicited nigrostriatal DA neuron damage and motor and non-motor deficits, providing a potential approach for the treatment of PD. Most neuropeptides could cross the blood-brain barrier (BBB) to play a neuroprotective role, such as ghrelin (Banks et al., 2002), NT (8–13) (Banks et al., 1995), PACAP (Banks et al., 1996), SP1–4 and SP3–11 (Freed et al., 2002); while NPY is poor to cross the BBB due to its lack of channels (Li et al., 2019).

Currently, there are several issues that need to be resolved. These issues would require further research into whether these changes in neuropeptide concentrations in CSF or plasma could be used as biomarkers. Additionally, it is needed to explore the potential use of analogs and receptor agonists in clinical trials for PD. Because there are extensive effects of these neuropeptides throughout the body, further studies are also needed to explore the side effects of neuropeptides and their analogs-related drugs in the treatment of PD.

## Author Contributions

YZ reviewed the literature, drafted, and revised the manuscript. LZ constructed the figures. JX revised the manuscript. LS conceived the study, revised the manuscript and provided funding support. All authors read and approved the final manuscript.

## Conflict of Interest

The authors declare that the research was conducted in the absence of any commercial or financial relationships that could be construed as a potential conflict of interest.

## References

[B1] Andrews Z. B. Erion D. Beiler R. Liu Z. W. Abizaid A. Zigman J. (2009). Ghrelin promotes and protects nigrostriatal dopamine function via a UCP2-dependent mitochondrial mechanism. *J. Neurosci.* 29 14057–14065. 10.1523/JNEUROSCI.3890-09.2009 19906954 PMC2845822

[B2] Antonelli T. Fuxe K. Tomasini M. C. Mazzoni E. Agnati L. F. Tanganelli S. (2007). Neurotensin receptor mechanisms and its modulation of glutamate transmission in the brain: relevance for neurodegenerative diseases and their treatment. *Prog. Neurobiol.* 83 92–109. 10.1016/j.pneurobio.2007.06.006 17673354

[B3] Banks W. Tschöp M. Robinson S. Heiman M. (2002). Extent and direction of ghrelin transport across the blood-brain barrier is determined by its unique primary structure. *J. Pharmacol. Exp. Ther.* 302 822–827. 10.1124/jpet.102.034827 12130749

[B4] Banks W. Uchida D. Arimura A. Somogyvári-Vigh A. Shioda S. (1996). Transport of pituitary adenylate cyclase-activating polypeptide across the blood-brain barrier and the prevention of ischemia-induced death of hippocampal neurons. *Ann. N. Y. Acad. Sci.* 805 270–277;discussion277–279. 10.1111/j.1749-6632.1996.tb17489.x 8993409

[B5] Banks W. Wustrow D. Cody W. Davis M. Kastin A. (1995). Permeability of the blood-brain barrier to the neurotensin8-13 analog NT1. *Brain Res.* 695 59–63. 10.1016/0006-8993(95)00836-f 8574648

[B6] Bayliss J. A. Andrews Z. B. (2013). Ghrelin is neuroprotective in Parkinson’s disease: molecular mechanisms of metabolic neuroprotection. *Ther. Adv. Endocrinol. Metab.* 4 25–36. 10.1177/2042018813479645 23515333 PMC3593299

[B7] Bayliss J. A. Lemus M. Santos V. V. Deo M. Elsworth J. D. Andrews Z. B. (2016a). Acylated but not des-acyl ghrelin is neuroprotective in an MPTP mouse model of Parkinson’s disease. *J. Neurochem.* 137 460–471. 10.1111/jnc.13576 26872221 PMC4836972

[B8] Bayliss J. A. Lemus M. B. Stark R. Santos V. V. Thompson A. Rees D. J. (2016b). Ghrelin-AMPK signaling mediates the neuroprotective effects of calorie restriction in Parkinson’s disease. *J. Neurosci.* 36 3049–3063. 10.1523/JNEUROSCI.4373-15.2016 26961958 PMC4783502

[B9] Brown D. Tamas A. Reglodi D. Tizabi Y. (2013). PACAP protects against salsolinol-induced toxicity in dopaminergic SH-SY5Y cells: implication for Parkinson’s disease. *J. Mol. Neurosci.* 50 600–607. 10.1007/s12031-013-0015-7 23625270 PMC3676705

[B10] Brown D. Tamas A. Reglodi D. Tizabi Y. (2014). PACAP protects against inflammatory-mediated toxicity in dopaminergic SH-SY5Y cells: implication for Parkinson’s disease. *Neurotox Res.* 26 230–239. 10.1007/s12640-014-9468-x 24740430

[B11] Caberlotto L. Fuxe K. Hurd Y. L. (2000). Characterization of NPY mRNA-expressing cells in the human brain: co-localization with Y2 but not Y1 mRNA in the cerebral cortex, hippocampus, amygdala, and striatum. *J. Chem. Neuroanat.* 20 327–337. 10.1016/s0891-0618(00)00107-1 11207429

[B12] Caberlotto L. Fuxe K. Rimland J. M. Sedvall G. Hurd Y. L. (1998). Regional distribution of neuropeptide Y Y2 receptor messenger RNA in the human post mortem brain. *Neuroscience* 86 167–178. 10.1016/s0306-4522(98)00039-6 9692752

[B13] Caberlotto L. Fuxe K. Sedvall G. Hurd Y. L. (1997). Localization of neuropeptide Y Y1 mRNA in the human brain: abundant expression in cerebral cortex and striatum. *Eur. J. Neurosci.* 9 1212–1225. 10.1111/j.1460-9568.1997.tb01476.x 9215705

[B14] Cannizzaro C. Tel B. C. Rose S. Zeng B. Y. Jenner P. (2003). Increased neuropeptide Y mRNA expression in striatum in Parkinson’s disease. *Brain Res. Mol. Brain Res.* 110 169–176. 10.1016/s0169-328x(02)00555-7 12591154

[B15] Carniglia L. Ramírez D. Durand D. Saba J. Turati J. Caruso C. (2017). Neuropeptides and microglial activation in inflammation, pain, and neurodegenerative diseases. *Mediators Inflamm.* 2017:5048616. 10.1155/2017/5048616 28154473 PMC5244030

[B16] Carraway R. Leeman S. E. (1973). The isolation of a new hypotensive peptide, neurotensin, from bovine hypothalami. *J. Biol. Chem.* 248 6854–6861. 4745447

[B17] Chang X. Ma Z. Shi L. Xie J. (2020). Effects of ghrelin on the electrical activities of substantia nigra dopaminergic neurons treated with MPP(.). *Neurochem. Int.* 138:104780. 10.1016/j.neuint.2020.104780 32569790

[B18] Chao O. Y. Wang A. L. Nikolaus S. de Souza Silva M. A. (2015). NK(3) receptor agonism reinstates temporal order memory in the hemiparkinsonian rat. *Behav. Brain Res.* 285 208–212. 10.1016/j.bbr.2014.06.006 24928770

[B19] Chen X. Y. Du Y. F. Chen L. (2018). Neuropeptides exert neuroprotective effects in Alzheimer’s disease. *Front. Mol. Neurosci.* 11:493. 10.3389/fnmol.2018.00493 30687008 PMC6336706

[B20] Chinaglia G. Probst A. Palacios J. M. (1990). Neurotensin receptors in Parkinson’s disease and progressive supranuclear palsy: an autoradiographic study in basal ganglia. *Neuroscience* 39 351–360. 10.1016/0306-4522(90)90273-7 1965015

[B21] Chu J. M. Chen L. W. Chan Y. S. Yung K. K. (2011). Neuroprotective effects of neurokinin receptor one in dopaminergic neurons are mediated through Akt/PKB cell signaling pathway. *Neuropharmacology* 61 1389–1398. 10.1016/j.neuropharm.2011.08.027 21907219

[B22] Chung C. Y. Seo H. Sonntag K. C. Brooks A. Lin L. Isacson O. (2005). Cell type-specific gene expression of midbrain dopaminergic neurons reveals molecules involved in their vulnerability and protection. *Hum. Mol. Genet.* 14 1709–1725. 10.1093/hmg/ddi178 15888489 PMC2674782

[B23] Cowley M. A. Smith R. G. Diano S. Tschop M. Pronchuk N. Grove K. L. (2003). The distribution and mechanism of action of ghrelin in the CNS demonstrates a novel hypothalamic circuit regulating energy homeostasis. *Neuron* 37 649–661. 10.1016/s0896-6273(03)00063-1 12597862

[B24] De Ceballos M. L. Lopez-Lozano J. J. (1999). Subgroups of parkinsonian patients differentiated by peptidergic immunostaining of caudate nucleus biopsies. *Peptides* 20 249–257. 10.1016/s0196-9781(98)00177-6 10422881

[B25] de Quidt M. E. Emson P. C. (1986). Distribution of neuropeptide Y-like immunoreactivity in the rat central nervous system–I. Radioimmunoassay and chromatographic characterisation. *Neuroscience* 18 527–543. 10.1016/0306-4522(86)90056-4 3755808

[B26] Decressac M. Pain S. Chabeauti P. Y. Frangeul L. Thiriet N. Herzog H. (2012). Neuroprotection by neuropeptide Y in cell and animal models of Parkinson’s disease. *Neurobiol. Aging* 33 2125–2137. 10.1016/j.neurobiolaging.2011.06.018 21816512

[B27] Deguil J. Chavant F. Lafay-Chebassier C. Perault-Pochat M. C. Fauconneau B. Pain S. (2010). Neuroprotective effect of PACAP on translational control alteration and cognitive decline in MPTP parkinsonian mice. *Neurotox Res.* 17 142–155. 10.1007/s12640-009-9091-4 19626386

[B28] Delgado M. Ganea D. (2003). Neuroprotective effect of vasoactive intestinal peptide (VIP) in a mouse model of Parkinson’s disease by blocking microglial activation. *FASEB J.* 17 944–946. 10.1096/fj.02-0799fje 12626429

[B29] Delhanty P. J. Neggers S. J. van der Lely A. J. (2012). Mechanisms in endocrinology: Ghrelin: the differences between acyl- and des-acyl ghrelin. *Eur. J. Endocrinol.* 167 601–608. 10.1530/EJE-12-0456 22898499

[B30] Dong J. Song N. Xie J. Jiang H. (2009). Ghrelin antagonized 1-methyl-4-phenylpyridinium (MPP(+))-induced apoptosis in MES23.5 cells. *J. Mol. Neurosci.* 37 182–189. 10.1007/s12031-008-9162-7 19052922

[B31] Duarte-Neves J. Goncalves N. Cunha-Santos J. Simoes A. T. den Dunnen W. F. Hirai H. (2015). Neuropeptide Y mitigates neuropathology and motor deficits in mouse models of Machado-Joseph disease. *Hum. Mol. Genet.* 24 5451–5463. 10.1093/hmg/ddv271 26220979

[B32] Feher M. Gaszner B. Tamas A. Gil-Martinez A. L. Fernandez-Villalba E. Herrero M. T. (2018). Alteration of the PAC1 receptor expression in the basal ganglia of MPTP-induced parkinsonian macaque monkeys. *Neurotox Res.* 33 702–715. 10.1007/s12640-017-9841-7 29230633

[B33] Fernandez A. de Ceballos M. L. Jenner P. Marsden C. D. (1994). Neurotensin, substance P, delta and mu opioid receptors are decreased in basal ganglia of Parkinson’s disease patients. *Neuroscience* 61 73–79. 10.1016/0306-4522(94)90061-2 7969897

[B34] Fernandez A. de Ceballos M. L. Rose S. Jenner P. Marsden C. D. (1996). Alterations in peptide levels in Parkinson’s disease and incidental Lewy body disease. *Brain* 119(Pt 3) 823–830. 10.1093/brain/119.3.823 8673494

[B35] Fernandez A. Jenner P. Marsden C. D. De Ceballos M. L. (1995). Characterization of neurotensin-like immunoreactivity in human basal ganglia: increased neurotensin levels in substantia nigra in Parkinson’s disease. *Peptides* 16 339–346. 10.1016/0196-9781(94)00141-3 7784265

[B36] Ferreira R. Xapelli S. Santos T. Silva A. P. Cristovao A. Cortes L. (2010). Neuropeptide Y modulation of interleukin-1{beta} (IL-1{beta})-induced nitric oxide production in microglia. *J. Biol. Chem.* 285 41921–41934. 10.1074/jbc.M110.164020 20959451 PMC3009919

[B37] Freed A. Audus K. Lunte S. J. P. (2002). Investigation of substance P transport across the blood-brain barrier. *Peptides* 23 157–165. 10.1016/s0196-9781(01)00592-7 11814631

[B38] Fumagalli F. Racagni G. Riva M. A. (2006). Shedding light into the role of BDNF in the pharmacotherapy of Parkinson’s disease. *Pharmacogenomics J.* 6 95–104. 10.1038/sj.tpj.6500360 16402079

[B39] Gong B. Jiao L. Du X. Li Y. Bi M. Jiao Q. (2020). Ghrelin promotes midbrain neural stem cells differentiation to dopaminergic neurons through Wnt/beta-catenin pathway. *J. Cell Physiol.* 235 8558–8570. 10.1002/jcp.29699 32329059

[B40] Goto S. Kawarai T. Morigaki R. Okita S. Koizumi H. Nagahiro S. (2013). Defects in the striatal neuropeptide Y system in X-linked dystonia-parkinsonism. *Brain* 136(Pt 5) 1555–1567. 10.1093/brain/awt084 23599389

[B41] Hashimoto H. Nogi H. Mori K. Ohishi H. Shigemoto R. Yamamoto K. (1996). Distribution of the mRNA for a pituitary adenylate cyclase-activating polypeptide receptor in the rat brain: an in situ hybridization study. *J. Comp. Neurol.* 371 567–577. 10.1002/(SICI)1096-9861(19960805)371:4<567::AID-CNE6<3.0.CO;2-2 8841910

[B42] Hou Z. Miao Y. Gao L. Pan H. Zhu S. (2006). Ghrelin-containing neuron in cerebral cortex and hypothalamus linked with the DVC of brainstem in rat. *Regul. Pept.* 134 126–131. 10.1016/j.regpep.2006.02.005 16600402

[B43] Jiang H. Li L. J. Wang J. Xie J. X. (2008). Ghrelin antagonizes MPTP-induced neurotoxicity to the dopaminergic neurons in mouse substantia nigra. *Exp. Neurol.* 212 532–537. 10.1016/j.expneurol.2008.05.006 18577498

[B44] Jiao Q. Du X. Li Y. Gong B. Shi L. Tang T. (2017). The neurological effects of ghrelin in brain diseases: beyond metabolic functions. *Neurosci. Biobehav. Rev.* 73 98–111. 10.1016/j.neubiorev.2016.12.010 27993602

[B45] Jomphe C. Lemelin P. L. Okano H. Kobayashi K. Trudeau L. E. (2006). Bidirectional regulation of dopamine D2 and neurotensin NTS1 receptors in dopamine neurons. *Eur. J. Neurosci.* 24 2789–2800. 10.1111/j.1460-9568.2006.05151.x 17116165

[B46] Kanazawa I. Jessell T. (1976). Post mortem changes and regional distribution of substance P in the rat and mouse nervous system. *Brain Res.* 117 362–367. 10.1016/0006-8993(76)90748-4 990924

[B47] Kerkerian L. Bosler O. Pelletier G. Nieoullon A. (1986). Striatal neuropeptide Y neurones are under the influence of the nigrostriatal dopaminergic pathway: immunohistochemical evidence. *Neurosci. Lett.* 66 106–112. 10.1016/0304-3940(86)90174-6 2872630

[B48] Kerkerian L. Salin P. Nieoullon A. (1988). Pharmacological characterization of dopaminergic influence on expression of neuropeptide Y immunoreactivity by rat striatal neurons. *Neuroscience* 26 809–817. 10.1016/0306-4522(88)90101-7 3143926

[B49] Kim W. Mohney R. Wilson B. Jeohn G. Liu B. Hong J. (2000). Regional difference in susceptibility to lipopolysaccharide-induced neurotoxicity in the rat brain: role of microglia. *J. Neurosci.* 20 6309–6316. 10.1523/jneurosci.20-16-06309.2000 10934283 PMC6772569

[B50] Kojima M. Hosoda H. Date Y. Nakazato M. Matsuo H. Kangawa K. (1999). Ghrelin is a growth-hormone-releasing acylated peptide from stomach. *Nature* 402 656–660. 10.1038/45230 10604470

[B51] Korkmaz O. Tunçel N. (2018). Advantages of vasoactive intestinal peptide for the future treatment of Parkinson’s disease. *Curr. Pharm. Des.* 24 4693–4701. 10.2174/1381612825666190111150953 30636594

[B52] Korkmaz O. Tunçel N. Tunçel M. Oncü E. Sahintürk V. Celik M. (2010). Vasoactive intestinal peptide (VIP) treatment of Parkinsonian rats increases thalamic gamma-aminobutyric acid (GABA) levels and alters the release of nerve growth factor (NGF) by mast cells. *J. Mol. Neurosci.* 41 278–287. 10.1007/s12031-009-9307-3 19953344

[B53] Lamine-Ajili A. Fahmy A. M. Letourneau M. Chatenet D. Labonte P. Vaudry D. (2016). Effect of the pituitary adenylate cyclase-activating polypeptide on the autophagic activation observed in *in vitro* and *in vivo* models of Parkinson’s disease. *Biochim. Biophys. Acta* 1862 688–695. 10.1016/j.bbadis.2016.01.005 26769362

[B54] Lazarova M. Popatanasov A. Klissurov R. Stoeva S. Pajpanova T. Kalfin R. (2018). Preventive effect of two new neurotensin analogues on Parkinson’s disease rat model. *J. Mol. Neurosci.* 66 552–560. 10.1007/s12031-018-1171-6 30374780

[B55] Lee D. Y. Hong S. H. Kim B. Lee D. S. Yu K. Lee K. S. (2018). Neuropeptide Y mitigates ER stress-induced neuronal cell death by activating the PI3K-XBP1 pathway. *Eur. J. Cell Biol.* 97 339–348. 10.1016/j.ejcb.2018.04.003 29650257

[B56] Li C. Wu X. Liu S. Zhao Y. Zhu J. Liu K. (2019). Roles of Neuropeptide Y in neurodegenerative and neuroimmune diseases. *Front. Neurosci.* 13:869. 10.3389/fnins.2019.00869 31481869 PMC6710390

[B57] Lindefors N. Brodin E. Tossman U. Segovia J. Ungerstedt U. (1989). Tissue levels and *in vivo* release of tachykinins and GABA in striatum and substantia nigra of rat brain after unilateral striatal dopamine denervation. *Exp. Brain Res.* 74 527–534. 10.1007/BF00247354 2468514

[B58] Liu L. Xu H. Jiang H. Wang J. Song N. Xie J. (2010). Ghrelin prevents 1-methyl-4-phenylpyridinium ion-induced cytotoxicity through antioxidation and NF-kappaB modulation in MES23.5 cells. *Exp. Neurol.* 222 25–29. 10.1016/j.expneurol.2009.11.009 19931250

[B59] Liu S. Chen S. Ren J. Li B. Qin B. (2018). Ghrelin protects retinal ganglion cells against rotenone via inhibiting apoptosis, restoring mitochondrial function, and activating AKT-mTOR signaling. *Neuropeptides* 67 63–70. 10.1016/j.npep.2017.11.007 29174113

[B60] Maasz G. Zrinyi Z. Reglodi D. Petrovics D. Rivnyak A. Kiss T. (2017). Pituitary adenylate cyclase-activating polypeptide (PACAP) has a neuroprotective function in dopamine-based neurodegeneration in rat and snail parkinsonian models. *Dis. Model Mech.* 10 127–139. 10.1242/dmm.027185 28067625 PMC5312006

[B61] Martignoni E. Blandini F. Petraglia F. Pacchetti C. Bono G. Nappi G. (1992). Cerebrospinal fluid norepinephrine, 3-methoxy-4-hydroxyphenylglycol and neuropeptide Y levels in Parkinson’s disease, multiple system atrophy and dementia of the Alzheimer type. *J. Neural. Transm. Park. Dis. Dement. Sect.* 4 191–205. 10.1007/BF02260903 1320891

[B62] Matus S. Glimcher L. H. Hetz C. (2011). Protein folding stress in neurodegenerative diseases: a glimpse into the ER. *Curr. Opin. Cell Biol.* 23 239–252. 10.1016/j.ceb.2011.01.003 21288706

[B63] Mauborgne A. Javoy-Agid F. Legrand J. C. Agid Y. Cesselin F. (1983). Decrease of substance P-like immunoreactivity in the substantia nigra and pallidum of parkinsonian brains. *Brain Res.* 268 167–170. 10.1016/0006-8993(83)90403-1 6190539

[B64] Minalyan A. Gabrielyan L. Pietra C. Tache Y. Wang L. (2019). Multiple beneficial effects of Ghrelin Agonist, HM01 on homeostasis alterations in 6-hydroxydopamine model of Parkinson’s disease in male rats. *Front. Integr. Neurosci.* 13:13. 10.3389/fnint.2019.00013 31031602 PMC6474391

[B65] Miyata A. Arimura A. Dahl R. R. Minamino N. Uehara A. Jiang L. (1989). Isolation of a novel 38 residue-hypothalamic polypeptide which stimulates adenylate cyclase in pituitary cells. *Biochem. Biophys. Res. Commun.* 164 567–574. 10.1016/0006-291x(89)91757-9 2803320

[B66] Moon M. Kim H. G. Hwang L. Seo J. H. Kim S. Hwang S. (2009). Neuroprotective effect of ghrelin in the 1-methyl-4-phenyl-1,2,3,6-tetrahydropyridine mouse model of Parkinson’s disease by blocking microglial activation. *Neurotox Res.* 15 332–347. 10.1007/s12640-009-9037-x 19384567

[B67] Morgan A. H. Rees D. J. Andrews Z. B. Davies J. S. (2018). Ghrelin mediated neuroprotection–A possible therapy for Parkinson’s disease? *Neuropharmacology* 136(Pt B) 317–326. 10.1016/j.neuropharm.2017.12.027 29277488

[B68] Nichol K. A. Morey A. Couzens M. H. Shine J. Herzog H. Cunningham A. M. (1999). Conservation of expression of neuropeptide Y5 receptor between human and rat hypothalamus and limbic regions suggests an integral role in central neuroendocrine control. *J. Neurosci.* 19 10295–10304. 10575027 10.1523/JNEUROSCI.19-23-10295.1999 PMC6782429

[B69] Obeso J. A. Rodriguez-Oroz M. C. Rodriguez M. Lanciego J. L. Artieda J. Gonzalo N. (2000). Pathophysiology of the basal ganglia in Parkinson’s disease. *Trends Neurosci. 23* 10(Suppl.) S8–S19. 10.1016/s1471-1931(00)00028-8 11052215

[B70] Obuchowicz E. Antkiewicz-Michaluk L. Romanska I. Herman Z. S. (2003). Increased striatal neuropeptide Y immunoreactivity and its modulation by deprenyl, clonidine and L-dopa in MPTP-treated mice. *J. Neural. Transm. (Vienna)* 110 1375–1391. 10.1007/s00702-003-0047-1 14666410

[B71] Pain S. Vergote J. Gulhan Z. Bodard S. Chalon S. Gaillard A. (2019). Inflammatory process in Parkinson disease: neuroprotection by neuropeptide Y. *Fundam. Clin. Pharmacol.* 33 544–548. 10.1111/fcp.12464 30866091

[B72] Pennefather J. N. Lecci A. Candenas M. L. Patak E. Pinto F. M. Maggi C. A. (2004). Tachykinins and tachykinin receptors: a growing family. *Life Sci.* 74 1445–1463. 10.1016/j.lfs.2003.09.039 14729395

[B73] Popelova A. Kakonova A. Hruba L. Kunes J. Maletinska L. Zelezna B. (2018). Potential neuroprotective and anti-apoptotic properties of a long-lasting stable analog of ghrelin: an in vitro study using SH-SY5Y cells. *Physiol. Res.* 67 339–346. 10.33549/physiolres.933761 29303606

[B74] Reglodi D. Kiss P. Lubics A. Tamas A. (2011). Review on the protective effects of PACAP in models of neurodegenerative diseases *in vitro* and *in vivo*. *Curr. Pharm. Des.* 17 962–972. 10.2174/138161211795589355 21524257

[B75] Reglodi D. Lubics A. Tamas A. Szalontay L. Lengvari I. (2004a). Pituitary adenylate cyclase activating polypeptide protects dopaminergic neurons and improves behavioral deficits in a rat model of Parkinson’s disease. *Behav. Brain Res.* 151 303–312. 10.1016/j.bbr.2003.09.007 15084446

[B76] Reglodi D. Renaud J. Tamas A. Tizabi Y. Socias S. B. Del-Bel E. (2017). Novel tactics for neuroprotection in Parkinson’s disease: role of antibiotics, polyphenols and neuropeptides. *Prog. Neurobiol.* 155 120–148. 10.1016/j.pneurobio.2015.10.004 26542398

[B77] Reglodi D. Tamas A. Lubics A. Szalontay L. Lengvari I. (2004b). Morphological and functional effects of PACAP in 6-hydroxydopamine-induced lesion of the substantia nigra in rats. *Regul. Pept.* 123 85–94. 10.1016/j.regpep.2004.05.016 15518897

[B78] Rivest R. St-Pierre S. Jolicoeur F. B. (1991). Structure-activity studies of neurotensin on muscular rigidity and tremors induced by 6-hydroxydopamine lesions in the posterolateral hypothalamus of the rat. *Neuropharmacology* 30 47–52. 10.1016/0028-3908(91)90041-9 1904561

[B79] Said S. I. Mutt V. (1970). Polypeptide with broad biological activity: isolation from small intestine. *Science* 169 1217–1218. 10.1126/science.169.3951.1217 5450698

[B80] Schimpff R. M. Avard C. Fenelon G. Lhiaubet A. M. Tenneze L. Vidailhet M. (2001). Increased plasma neurotensin concentrations in patients with Parkinson’s disease. *J. Neurol. Neurosurg. Psychiatry* 70 784–786. 10.1136/jnnp.70.6.784 11385014 PMC1737416

[B81] Schroder J. B. Marian T. Claus I. Muhle P. Pawlowski M. Wiendl H. (2019). Substance P Saliva reduction predicts pharyngeal dysphagia in Parkinson’s disease. *Front. Neurol.* 10:386. 10.3389/fneur.2019.00386 31040820 PMC6477048

[B82] Sendtner M. Holtmann B. Kolbeck R. Thoenen H. Barde Y. A. (1992). Brain-derived neurotrophic factor prevents the death of motoneurons in newborn rats after nerve section. *Nature* 360 757–759. 10.1038/360757a0 1465147

[B83] Shi L. Bian X. Qu Z. Ma Z. Zhou Y. Wang K. (2013). Peptide hormone ghrelin enhances neuronal excitability by inhibition of Kv7/KCNQ channels. *Nat. Commun.* 4:1435. 10.1038/ncomms2439 23385580

[B84] Shi L. Du X. Jiang H. Xie J. (2017). Ghrelin and neurodegenerative disorders-a review. *Mol. Neurobiol.* 54 1144–1155. 10.1007/s12035-016-9729-1 26809582

[B85] Shivers K. Y. Nikolopoulou A. Machlovi S. I. Vallabhajosula S. Figueiredo-Pereira M. E. (2014). PACAP27 prevents Parkinson-like neuronal loss and motor deficits but not microglia activation induced by prostaglandin J2. *Biochim. Biophys. Acta* 1842 1707–1719. 10.1016/j.bbadis.2014.06.020 24970746 PMC4125523

[B86] Sivam S. P. (1991). Dopamine dependent decrease in enkephalin and substance P levels in basal ganglia regions of postmortem parkinsonian brains. *Neuropeptides* 18 201–207. 10.1016/0143-4179(91)90148-c 1711165

[B87] Song N. Wang W. Jia F. Du X. Xie A. He Q. (2017). Assessments of plasma ghrelin levels in the early stages of parkinson’s disease. *Mov. Disord.* 32 1487–1491. 10.1002/mds.27095 28681931

[B88] Starback P. Wraith A. Eriksson H. Larhammar D. (2000). Neuropeptide Y receptor gene y6: multiple deaths or resurrections? *Biochem. Biophys. Res. Commun.* 277 264–269. 10.1006/bbrc.2000.3656 11027673

[B89] Stoddard S. L. Tyce G. M. Ahlskog J. E. Zinsmeister A. R. Nelson D. K. Carmichael S. W. (1991). Decreased levels of [Met]enkephalin, neuropeptide Y, substance P, and vasoactive intestinal peptide in parkinsonian adrenal medulla. *Exp. Neurol.* 114 23–27. 10.1016/0014-4886(91)90080-v 1915731

[B90] Suda Y. Kuzumaki N. Sone T. Narita M. Tanaka K. Hamada Y. (2018). Down-regulation of ghrelin receptors on dopaminergic neurons in the substantia nigra contributes to Parkinson’s disease-like motor dysfunction. *Mol. Brain* 11:6. 10.1186/s13041-018-0349-8 29458391 PMC5819262

[B91] Svenningsson P. Palhagen S. Mathe A. A. (2017). Neuropeptide Y and calcitonin gene-related peptide in cerebrospinal fluid in Parkinson’s disease with comorbid depression versus patients with major depressive disorder. *Front. Psychiatry* 8:102. 10.3389/fpsyt.2017.00102 28659833 PMC5466951

[B92] Tatemoto K. (1982). Neuropeptide Y: complete amino acid sequence of the brain peptide. *Proc. Natl. Acad. Sci. U.S.A.* 79 5485–5489. 10.1073/pnas.79.18.5485 6957876 PMC346928

[B93] Tenovuo O. Rinne U. K. Viljanen M. K. (1984). Substance P immunoreactivity in the post-mortem parkinsonian brain. *Brain Res.* 303 113–116. 10.1016/0006-8993(84)90217-8 6203617

[B94] Thornton E. Hassall M. M. Corrigan F. Vink R. (2014). The NK1 receptor antagonist N-acetyl-L-tryptophan reduces dyskinesia in a hemi-parkinsonian rodent model. *Parkinsonism Relat. Disord.* 20 508–513. 10.1016/j.parkreldis.2014.02.008 24637127

[B95] Thornton E. Vink R. (2012). Treatment with a substance P receptor antagonist is neuroprotective in the intrastriatal 6-hydroxydopamine model of early Parkinson’s disease. *PLoS One* 7:e34138. 10.1371/journal.pone.0034138 22485158 PMC3317489

[B96] Tunçel N. Korkmaz O. Tekin N. Şener E. Akyüz F. Inal M. (2012). Antioxidant and anti-apoptotic activity of vasoactive intestinal peptide (VIP) against 6-hydroxy dopamine toxicity in the rat corpus striatum. *J. Mol. Neurosci.* 46 51–57. 10.1007/s12031-011-9618-z 21850490

[B97] Tunçel N. Sener E. Cerit C. Karasu U. Gürer F. Sahintürk V. (2005). Brain mast cells and therapeutic potential of vasoactive intestinal peptide in a Parkinson’s disease model in rats: brain microdialysis, behavior, and microscopy. *Peptides* 26 827–836. 10.1016/j.peptides.2004.12.019 15808913

[B98] Uhl G. R. Whitehouse P. J. Price D. L. Tourtelotte W. W. Kuhar M. J. (1984). Parkinson’s disease: depletion of substantia nigra neurotensin receptors. *Brain Res.* 308 186–190. 10.1016/0006-8993(84)90935-1 6089953

[B99] Vaudry D. Falluel-Morel A. Bourgault S. Basille M. Burel D. Wurtz O. (2009). Pituitary adenylate cyclase-activating polypeptide and its receptors: 20 years after the discovery. *Pharmacol. Rev.* 61 283–357. 10.1124/pr.109.001370 19805477

[B100] Vincent J. P. Mazella J. Kitabgi P. (1999). Neurotensin and neurotensin receptors. *Trends Pharmacol. Sci.* 20 302–309. 10.1016/s0165-6147(99)01357-7 10390649

[B101] Wagner J. Vulinovic F. Grunewald A. Unger M. M. Moller J. C. Klein C. (2017). Acylated and unacylated ghrelin confer neuroprotection to mesencephalic neurons. *Neuroscience* 365 137–145. 10.1016/j.neuroscience.2017.09.045 28987508

[B102] Wang G. Pan J. Tan Y. Y. Sun X. K. Zhang Y. F. Zhou H. Y. (2008). Neuroprotective effects of PACAP27 in mice model of Parkinson’s disease involved in the modulation of K(ATP) subunits and D2 receptors in the striatum. *Neuropeptides* 42 267–276. 10.1016/j.npep.2008.03.002 18440632

[B103] Wang G. Qi C. Fan G. H. Zhou H. Y. Chen S. D. (2005). PACAP protects neuronal differentiated PC12 cells against the neurotoxicity induced by a mitochondrial complex I inhibitor, rotenone. *FEBS Lett.* 579 4005–4011. 10.1016/j.febslet.2005.06.013 16004991

[B104] Wang Q. Chu C. H. Qian L. Chen S. H. Wilson B. Oyarzabal E. (2014). Substance P exacerbates dopaminergic neurodegeneration through neurokinin-1 receptor-independent activation of microglial NADPH oxidase. *J. Neurosci.* 34 12490–12503. 10.1523/JNEUROSCI.2238-14.2014 25209287 PMC4160779

[B105] Wang Q. Oyarzabal E. Wilson B. Qian L. Hong J. S. (2015a). Substance P enhances microglial density in the substantia nigra through neurokinin-1 receptor/NADPH oxidase-mediated chemotaxis in mice. *Clin. Sci. (Lond)* 129 757–767. 10.1042/CS20150008 26223840 PMC5464607

[B106] Wang S. Y. Chen L. Xue Y. Xia Y. J. (2015b). Substance P prevents 1-methyl-4-phenylpyridinium-induced cytotoxicity through inhibition of apoptosis via neurokinin-1 receptors in MES23.5 cells. *Mol. Med. Rep.* 12 8085–8092. 10.3892/mmr.2015.4464 26497672

[B107] Xue B. Li C. Chang X. Jiang H. Shi L. Xie J. (2020). Ghrelin Reduces A-type potassium currents in dopaminergic nigral neurons via the PLC/PKCdelta pathway. *Neurosci. Bull.* 36 947–950. 10.1007/s12264-020-00508-4 32388709 PMC7410936

[B108] Yamada M. Yamada M. Richelson E. (1995). Heterogeneity of melanized neurons expressing neurotensin receptor messenger RNA in the substantia nigra and the nucleus paranigralis of control and Parkinson’s disease brain. *Neuroscience* 64 405–417. 10.1016/0306-4522(94)00395-l 7700529

[B109] Yan H. Yang J. Marasco J. Yamaguchi K. Brenner S. Collins F. (1996). Cloning and functional expression of cDNAs encoding human and rat pancreatic polypeptide receptors. *Proc. Natl. Acad. Sci. U.S.A.* 93 4661–4665. 10.1073/pnas.93.10.4661 8643460 PMC39335

[B110] Yang S. Yang J. Yang Z. Chen P. Fraser A. Zhang W. (2006). Pituitary adenylate cyclase-activating polypeptide (PACAP) 38 and PACAP4-6 are neuroprotective through inhibition of NADPH oxidase: potent regulators of microglia-mediated oxidative stress. *J. Pharmacol. Exp. Ther.* 319 595–603. 10.1124/jpet.106.102236 16891616

[B111] Yang X. Zhao H. Shi H. Wang X. Zhang S. Zhang Z. (2015). Intranigral administration of substance P receptor antagonist attenuated levodopa-induced dyskinesia in a rat model of Parkinson’s disease. *Exp. Neurol.* 271 168–174. 10.1016/j.expneurol.2015.05.007 26001615

[B112] Yu J. Xu H. Shen X. Jiang H. (2016). Ghrelin protects MES23.5 cells against rotenone via inhibiting mitochondrial dysfunction and apoptosis. *Neuropeptides* 56 69–74. 10.1016/j.npep.2015.09.011 26459609

[B113] Yu R. Li J. Lin Z. Ouyang Z. Huang X. Reglodi D. (2020). TAT-tagging of VIP exerts positive allosteric modulation of the PAC1 receptor and enhances VIP neuroprotective effect in the MPTP mouse model of Parkinson’s disease. *Biochim. Biophys. Acta Gen. Subj.* 1864:129626. 10.1016/j.bbagen.2020.129626 32335135

